# Supplementary material for: Gate-reflectometry dispersive readout and coherent control of a spin qubit in silicon
Source: Nat Commun. 2019 Jul 3;10:2776. doi: 10.1038/s41467-019-10848-z (PMC6610084; doi:10.1038/s41467-019-10848-z)
Supplement: Supplementary file 1 — Supplementary Information [file 41467_2019_10848_MOESM1_ESM.pdf]

Supplementary Information: Gate-reflectometry dispersive readout and coherent control of a spin qubit in silicon

A. Crippa,[1,*] R. Ezzouch,[1] A. Aprá,[1] A. Amisse,[1] R. Laviéville,[2] L. Hutin,[2] B. Bertrand,[2] M. Vinet,[2] M. Urdampilleta,[3] T. Meunier,[3] M. Sanquer,[1] X. Jehl,[1] R. Maurand,[1] and S. De Franceschi[1]

[1] *Univ. Grenoble Alpes, CEA, INAC-PHELIQS, F-38000 Grenoble, France*
[2] *CEA, LETI, Minatec Campus, F-38000 Grenoble, France*
[3] *Univ. Grenoble Alpes, CNRS, Grenoble INP, Institut Néel, F-38000 Grenoble, France*

SUPPLEMENTARY NOTE 1: MEASUREMENT CIRCUIT

Supplementary Figure 1 shows the measurement circuitry of the experiment.
DC voltages are generated by room-temperature digital-to-analogue converters and filtered at low temperatures by home-made silver epoxy filters and 2-stage RC filters. These signals are applied to the two gate electrodes and source and drain contacts, the latter kept at 0 mV throughout the whole experiment.
The reflectometry channel is fed by an Agilent N5181A RF source, which also provides the reference signal of a Zurich Ultra-High Frequency (UHF) lock-in for demodulation. The reflectometry tone is pass-band filtered at room temperature and attenuated at different stages of the fridge. It is added to the DC signal via a bias tee mounted on the sample holder. The tank circuit consists in a 220 nH surface-mount inductor (Coilcraft 221XGLB) and a parasitic capacitance. The reflected signal is separated from the incoming wave by a directional coupler and amplified at the 4 K stage.
One output of the UHF is used in the AWG mode to precisely gate the microwave tone delivered by an Agilent E8257D source for coherent spin rotations. The resulting microwave bursts are added by a triplexer to the read-out/manipulation pulses generated by the other UHF channel. The signal then passes through different attenuators and feeds a bias tee on the board.

SUPPLEMENTARY NOTE 2: LARGE STABILITY DIAGRAM

Supplementary Figure 2 shows two stability diagrams of the device under investigation. Both plots share the same V_C voltage range. The other gate tunes the electrostatics of the channel from the many hole regime (bottom panel) to the voltage region we use to implement our qubit (top panel). In particular, the blue square highlights the area zoomed in the stability map of Fig. 2a in the main text. Considering the diagram as a whole, two sets of features are present. First, a series of nearly horizontal parallel lines are visible. These lines repeat quite regularly from metallic DQDs to depletion, even the silicon channel is completely closed (data not shown). Consequently, we speculate that these features are related to the charging of objects extrinsic to the channel.
On top of this background, most of the short diagonal cuts on the yellow background are interdot transition lines. The bottom part of Supplementary Fig. 2 reports the many hole regime where the voltage spacing between DQDs is approximately constant. The typical gate voltage between two charge states is about 25 mV. This value is consistent with other experiments on similar samples [1, 2]. Out of the many hole regime, the interdot lines are unevenly spaced, as displayed in the top panel. Importantly, for interdot tunnel couplings of few GHz (like the one studied in the main text), the interdot transition lines are quite thin in gate voltage, and are very likely not resolved in large maps obtained with large voltage steps. We use the threshold voltages at room temperature of the two gates and the addition voltage of the many hole regime for a rough estimation of the absolute filling of the dots. We obtain an order of magnitude of 5 holes and 10-20 holes in the left (i.e. mainly controlled by V_R) and right dot (mainly controlled by V_C), respectively.

SUPPLEMENTARY NOTE 3: DISPERSIVE RESPONSE OF THE DQD

Model

In this Section we derive the spectrum the hole DQD close to the interdot charge transition presented in Fig. 2a. As discussed in the main text, the dispersive signal is attributed to $(''1,1'') \leftrightarrow (''0,2'')$ charge transfers. The superscripts

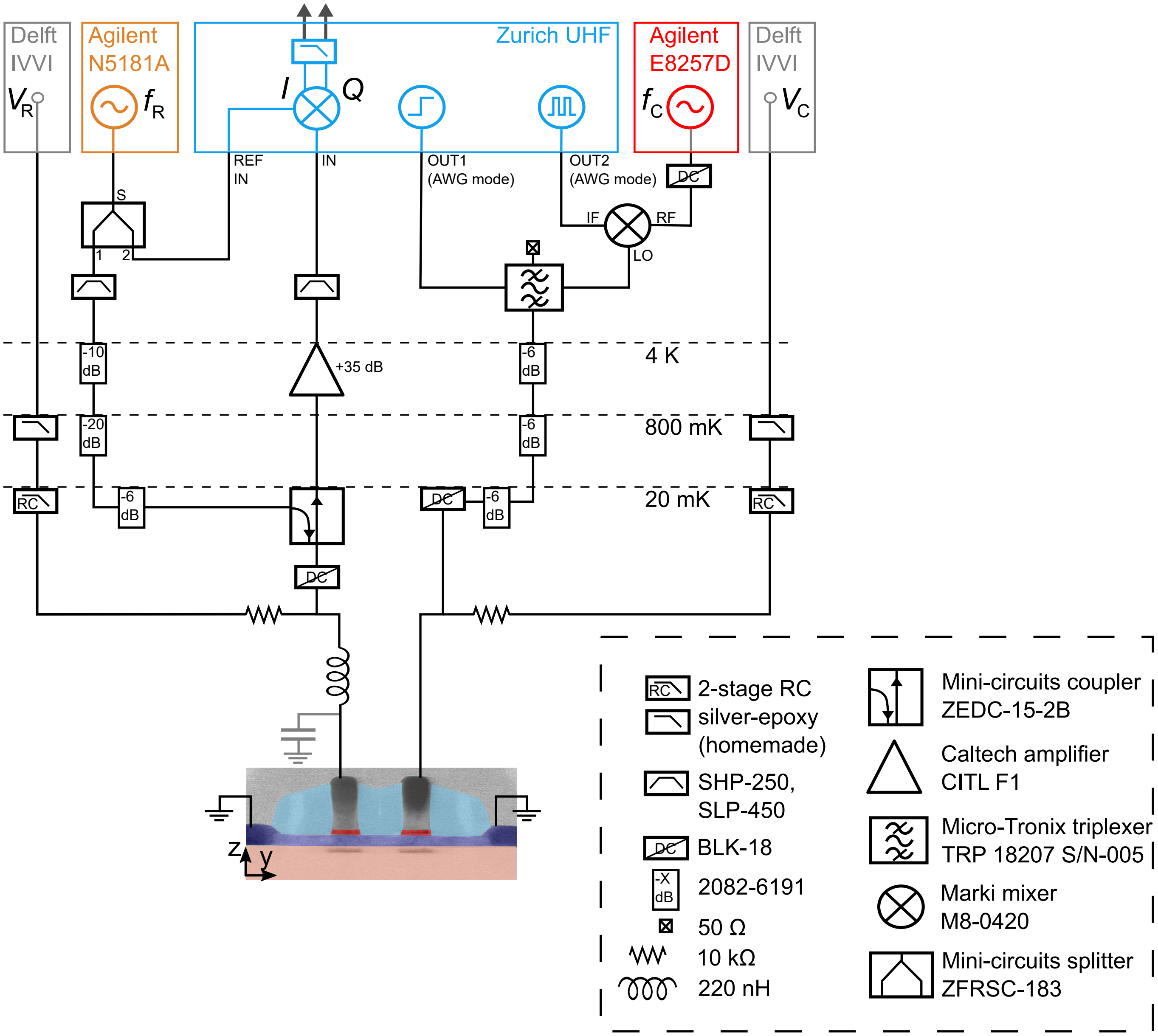

Supplementary Figure 1. Schematic of the qubit measurement setup. The circuitry of the right gate combines DC voltages for DQD electrostatic tuning, fast voltage pulses and EDSR microwave tones. For the left gate, the DC voltage is added to reflectometry radiofrequency signal for dispersive homodyne detection.

" indicate that the numbers denote the parity-equivalent excess holes of the double dot.
The excess charge of each dot is a qubit with spin-orbit eigenstates $|\Uparrow\rangle$ and $|\Downarrow\rangle$. The external magnetic field B induces a Zeeman splitting between $|\Downarrow\rangle$ and $|\Uparrow\rangle$ equal to $g^*_{L(R)}\mu_B B$, with $g^*_{L(R)}$ the effective g-factor of the left (right) dot for a given direction of B and μ_B the Bohr magneton.
We describe the DQD through the lowest five energy states in the basis $\{|\Uparrow\Uparrow\rangle, |\Uparrow\Downarrow\rangle, |\Downarrow\Uparrow\rangle, |\Downarrow\Downarrow\rangle, \frac{1}{\sqrt{2}}|0\,(\Uparrow\Downarrow - \Downarrow\Uparrow)\rangle\}$.
The orbital spacing is $\sim 1\,\mathrm{meV}$, which allows us to neglect the excited (triplet-like) $(0,2)$ state. The Hamiltonian

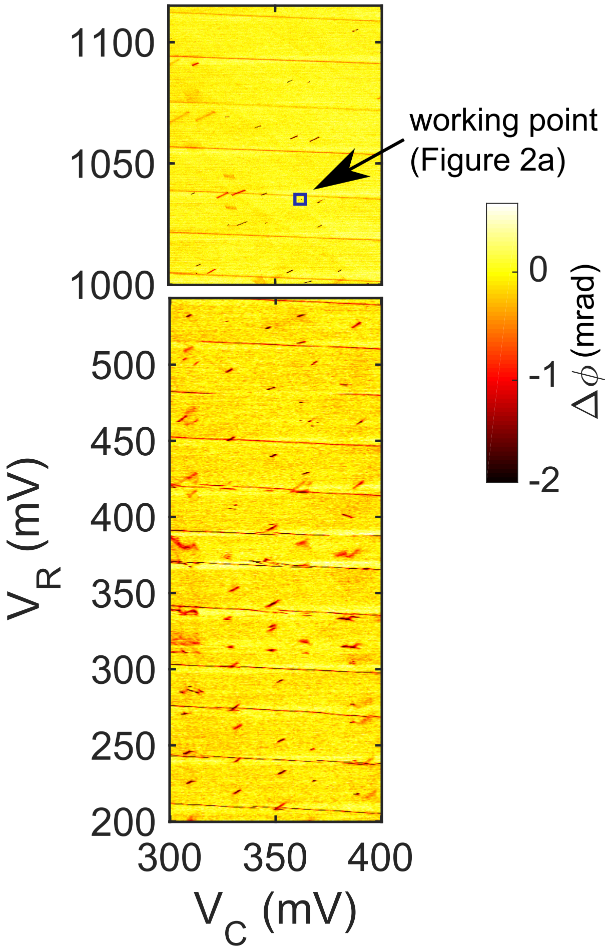

Supplementary Figure 2. Dispersively detected charge stability diagram of the device as a function of the two top gate voltages, V_C and V_R. In the bottom panel, both gates are tuned in a strong accumulation mode, and the many hole regime, characterized by a regular arrangement of the interdot transition lines, is reached. In the upper panel, V_R approaches the gate voltage threshold; as a result, interdot charge transitions are unequally placed. The blue square denotes the area zoomed-in in Fig. 2a of the main text.

then reads:

$$H = \begin{pmatrix} -\frac{1}{2}\epsilon + \frac{1}{2}(g_L^* + g_R^*)\mu_B B & 0 & 0 & 0 & 0 \\ 0 & -\frac{1}{2}\epsilon + \frac{1}{2}(g_L^* - g_R^*)\mu_B B & 0 & 0 & \frac{t}{\sqrt{2}} \\ 0 & 0 & -\frac{1}{2}\epsilon - \frac{1}{2}(g_L^* - g_R^*)\mu_B B & 0 & -\frac{t}{\sqrt{2}} \\ 0 & 0 & 0 & -\frac{1}{2}\epsilon - \frac{1}{2}(g_L^* + g_R^*)\mu_B B & 0 \\ 0 & \frac{t}{\sqrt{2}} & -\frac{t}{\sqrt{2}} & 0 & \frac{1}{2}\epsilon \end{pmatrix}. \tag{1}$$

In this expression the DQD detuning ϵ is with respect to the middle point energy between the eigenstates at $B = 0\,\mathrm{T}$. The tunnel coupling t connects the antiparallel spin states $|\Downarrow\Uparrow\rangle$ and $|\Uparrow\Downarrow\rangle$ to the singlet $S(0,2) = \frac{1}{\sqrt{2}}\,|0\,(\Uparrow\Downarrow - \Downarrow\Uparrow)\rangle$, thereby allowing interdot charge transitions.

The DQD spectrum in Fig. 2d of the main text displays the eigenvalues of Supplementary Eq. 1 as a function of ϵ with $g_L^* = 1.62$, $g_R^* = 2.12$, $t = 8\,\mu\mathrm{eV}$ and $B = 0.65\,\mathrm{T}$.

DQD dispersive response

To clarify the origin of the dispersive signal close to the interdot transition line, we map the DQD of Supplementary Eq. 1 onto a singlet-triplet basis $\{T_+(1,1), T_0(1,1), T_-(1,1), S(1,1), S(0,2)\}$:

$$H' = \begin{pmatrix} -\frac{1}{2}\epsilon + \frac{1}{2}(g_L^* + g_R^*)\mu_B B & 0 & 0 & 0 & 0 \\ 0 & -\frac{1}{2}\epsilon & 0 & \frac{1}{2}(g_L^* - g_R^*)\mu_B B & 0 \\ 0 & 0 & -\frac{1}{2}\epsilon - \frac{1}{2}(g_L^* + g_R^*)\mu_B B & 0 & 0 \\ 0 & \frac{1}{2}(g_L^* - g_R^*)\mu_B B & 0 & -\frac{1}{2}\epsilon & t \\ 0 & 0 & 0 & t & \frac{1}{2}\epsilon \end{pmatrix}. \quad (2)$$

The singlet states have a curvature due to coupling term t, which leads to a non-zero quantum capacitance and a consequent dispersive shift of the resonant frequency. Concerning $T_0(1,1)$, it is usually not dispersively sensed since its second derivative with respect to ϵ is zero. However, Fig. 2e shows a finite phase response for $T_0(1,1)$. It comes from the electric dipole due to the coupling with $S(1,1)$ via $\frac{1}{2}(g_L^* - g_R^*)\mu_B B$, which eventually implies a second-order coupling with $S(0,2)$.

We model the DQD dispersive response by quantum capacitance contributions with a Boltzmann distribution [3, 4]. Fast excitations/relaxations in the singlet manifold may contribute to the DQD phase response through the tunnel capacitance [3]. However, if such nonadiabatic processes are slow (~ 100 MHz) compared to the probing frequency f_R, the tunnel capacitance is small with respect to the quantum capacitance; on the other hand, with fast charge relaxations (~ 1 GHz) the interdot ridge would have lineshape and width not compatible with the magnetic field evolution reported in Fig. 2.

In the basis set of Supplementary Eq. 2, the spin-orbit (SO) transition matrix elements are supposed weak compared to t and the Zeeman terms. Sizable spin-flip tunnelling terms like $t_{SO}^{|T_-\rangle} |T_-(1,1)\rangle \langle S(0,2)|$ and $t_{SO}^{|T_+\rangle} |T_+(1,1)\rangle \langle S(0,2)|$ would lead to a dispersive signal with a strong magnetic field dependence. We found no evidence of the corresponding dispersive signals in the magnetospectroscopy data discussed in the main text.
A coupling factor $t_{SO}^{|T_0(1,1)\rangle}$ between $T_0(1,1)$ and $S(0,2)$ comparable to t has not to be expected neither. From simulations at $B > 0.5$ T, with $t_{SO}^{|T_0\rangle} \sim t$ the phase resonance of the interdot transitions would resemble a pronounced peak with a bearly-visible shoulder on the right edge, not consistent with data in Figs. 2 and 3 of the main text. However, we can't rule out these such spin-flip tunneling terms might be relevant for orientations of the external magnetic field different from those investigated here.

A comprehensive description of the experimental phase signal is achieved by considering the excited levels of the DQD as partially populated. Each state leads to an averaged phase signal $\langle \Delta\phi \rangle_i = \Delta\phi_i^{T=0} \cdot e^{-E_i \beta}/Z$, where $\Delta\phi_i^{T=0}$ is proportional to the quantum capacitance of the state with energy E_i in the 0 K limit, $\beta = 1/k_B T_{\mathrm{eff}}$ with k_B Boltzmann constant and T_{eff} an effective temperature, and Z is the partition function over the five states. The measured phase signal then is $\Delta\phi = \sum_i \langle \Delta\phi \rangle_i$.
The coupling term t is estimated from a detuning trace at $B = 0$ T. According to the model just described, the full width at half maximum (FWHM) of the phase interdot ridge as a function of the effective temperature T_{eff} evolves as shown in Supplementary Figure 3.

Two limiting situations are envisaged. At low temperature, $k_B T_{\mathrm{eff}} < t/10$ and the width of the interdot signal is set by the tunnel coupling to $\sim 3t$. Here just the ground singlet is populated. In the opposite limit of high temperature, $k_B T_{\mathrm{eff}} > 2t$, the threefold triplet and both bonding and anti-bonding singlet are thermally populated; by sweeping T_{eff}, the magnitude of the interdot resonance drops, but the FWHM saturates at $\sim 4t$. In the intermediate regime, the FWHM increases progressively with T_{eff}, up to the saturation point occurring at $k_B T_{\mathrm{eff}} \simeq 2t$.
Furthermore, Supplementary Fig. 3 demonstrates that the FWHM allows to estimate t in the $(3t, 4t)$ range whatever the temperature is. This distinguishes dispersive readout from charge sensing (especially when $k_B T_{\mathrm{eff}} > 2t$), as the resonator sensitivity is ultimately constrained to the avoided crossings in the energy level diagram.
Fits to the interdot detuning phase shift yield $t = 8.5\,\mu$eV and $t = 6.4\,\mu$eV in the low and high temperature limit, respectively. The evolution of the interdot transition line versus the magnetic field is reproduced qualitatively assuming the lowest tunnel coupling and 0.25 K as effective temperature. The inset in Fig. 2b of the main text is obtained with $g_L^* = 1.52$, $g_R^* = 2.02$, $t = 6\,\mu$eV and $T_{\mathrm{eff}} = 0.25$ K. The one-dimensional cuts in Fig. 2f are taken at $B = 0$, 0.35, 0.5 and 0.85 T.

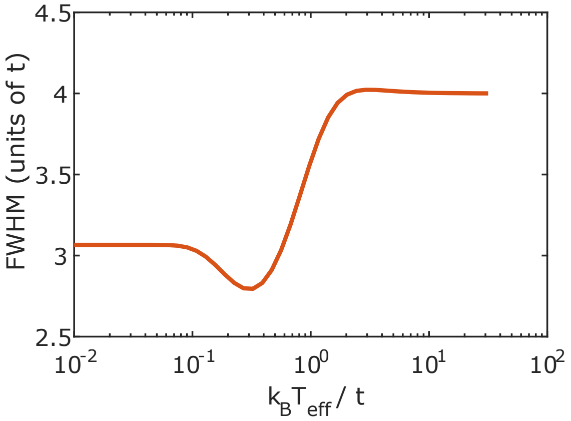

Supplementary Figure 3. Predicted evolution of the full width at half maximum (FWHM) of the interdot dispersive resonance as a function of temperature at $B = 0\,\mathrm{T}$. The phase signal is supposed composed solely of quantum capacitance contributions.

SUPPLEMENTARY NOTE 4: DETUNING POSITION FOR DISPERSIVE READOUT

Inset of Fig. 3c shows the behavior of the phase signal we expect when second harmonic EDSR transitions are promoted between the $|T_-\rangle$ and $|T_0\rangle$ states at point I/R (we write the states as kets from now on). From our model, we set $g_L^* = 1.575$, $g_R^* = 2.075$, $t = 6\,\mu\mathrm{eV}$, $B = 0.52\,\mathrm{T}$ and $T_{\mathrm{eff}} = 0.25\,\mathrm{K}$. We also impose $f_C = 7.42\,\mathrm{GHz}$, as in the experimental trace of the main panel. We find that the resonant condition $f_C = |E_{|T_0\rangle} - E_{|T_-\rangle}|/2h$ is met at finite ϵ ($23.5\,\mu\mathrm{eV}$), in agreement with data. At this detuning, we model the EDSR peak by equalizing the occupation probability of both states to $[P_{|T_-\rangle} + P_{|T_0\rangle}]/2$, where $P_{|T_-\rangle} = \exp(-E_{|T_-\rangle}\beta)/Z$ and $P_{|T_0\rangle} = \exp(-E_{|T_0\rangle}\beta)/Z$. The phase response is then convolved with a Gaussian distribution with a variance of $50\,(\mu\mathrm{eV})^2$ accounting for the observed detuning broadening. One might expect the detuning position of the EDSR peak to depend on f_C, along with an increase of phase signal with approaching to $\epsilon = 0$. However, as observed in other types of Si qubits [5–7], in the vicinity of $\epsilon = 0$ decoherence rates increase as well, which limits the detuning window for convenient reflectometry readout.

Supplementary Figure 4 shows the phase response as a function of detuning (along V_C) and EDSR driving frequency. The B field is oriented as in the qubit measurements of Fig. 4 of the main text. The phase signal due to EDSR transitions is highlighted by the orange arrow of panel b), while the other nonzero phase features are due to spurious photon-assisted events or noise.

As pointed out in the main text, the microwave-induced population of $|T_0\rangle$ state is detected in a "shallow" $(1,1)$ charge stability region ($\epsilon > 0.02\,\mathrm{meV}$) with a nearly constant dispersion $d|E_{|T_0\rangle} - E_{|T_-\rangle}|/d\epsilon$. In this regime, the qubit is robust with respect to fluctuations in the energy splitting, as testified by the relaxation time in the microsecond range. Closer to the alignment of the electrochemical potentials of the two dots ($0 < \epsilon < 0.02\,\mathrm{meV}$), the EDSR signal is not resolved, probably due to inhomogeneous energy broadening between the resonant states. This makes this bias regime unsuitable for readout.

SUPPLEMENTARY NOTE 5: T_1 ESTIMATION

To extract the spin relaxation time at the readout position I of Fig. 4b, we use a pulse length of $250\,\mathrm{ns}$ and sweep t_{wait}, see Fig. 4a. During the pulse, a microwave burst of $100\,\mathrm{ns}$ flips one of the two spins. We normalize the amplitude of the phase shift by a factor $(1 + 250\,\mathrm{ns}/t_{\mathrm{wait}})$ since the signal is acquired during the whole period T_M.

The readout projects $|\Uparrow\Downarrow\rangle$ on the $\{|T_0\rangle, |T_-\rangle\}$ basis. The time-dependent probability that the spin relaxes in $|T_-\rangle$ is given by $P(t)_{|T_-\rangle} = P(t=0)_{|T_0\rangle} \exp(-t/T_1)$. The time averaged data points in Fig. 4b are then fitted to $\Delta\phi = a_0 - a_1 \frac{T_1}{T_M}\big(\exp(-T_M/T_1) - 1\big)$, with $T_M = t_{\mathrm{wait}} + 250\,\mathrm{ns}$.

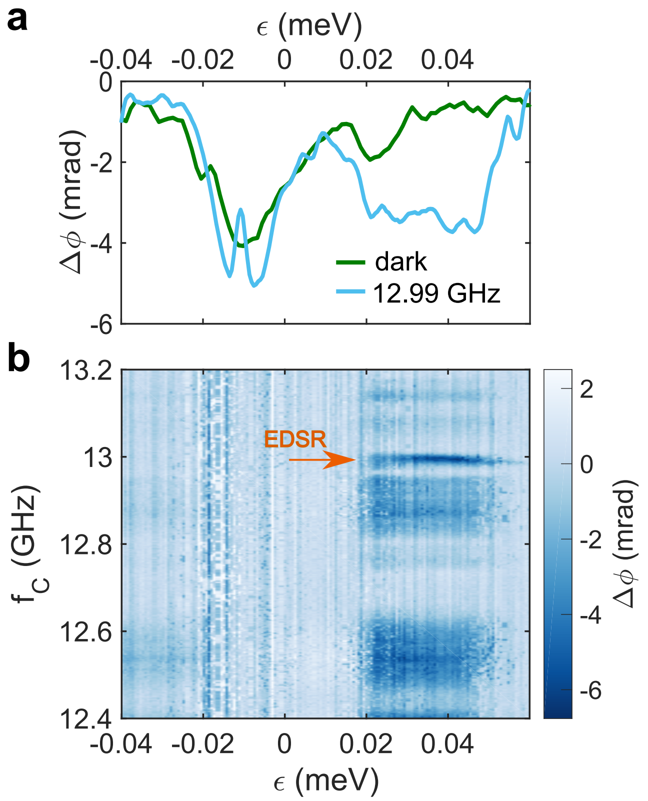

Supplementary Figure 4. (a) One-dimensional detuning scan of the interdot line without microwave radiation ('dark') and with $f_C = 12.99\,\mathrm{GHz}$ applied. The right peak (associated to $|T_0\rangle$) is enhanced and largely broadened when EDSR transitions take place. (b) Colorplot of the phase response as a function of the detuning and the driving frequency. The map is acquired by sweeping f_C and stepping ϵ; at the beginning of each line, the phase of the reflectometry signal is set to 0. Signal related to EDSR is indicated by the orange arrow.

* alessandro.crippa@cea.fr or romain.maurand@cea.fr

[1] R. Maurand, X. Jehl, D. Kotekar-Patil, A. Corna, H. Bohuslavskyi, R. Laviéville, L. Hutin, S. Barraud, M. Vinet, M. Sanquer, and S. De Franceschi, Nature Comm. **7**, 13575 (2016).
[2] A. Crippa, R. Maurand, L. Bourdet, D. Kotekar-Patil, A. Amisse, X. Jehl, M. Sanquer, R. Laviéville, H. Bohuslavskyi, L. Hutin, S. Barraud, M. Vinet, Y. Niquet, and S. De Franceschi, Phys. Rev. Lett. **120**, 137702 (2018).
[3] R. Mizuta, R. Otxoa, A. Betz, and M. F. Gonzalez-Zalba, Phys. Rev. B **95**, 045414 (2017).
[4] M. Schroer, M. Jung, K. Petersson, and J. Petta, Phys. Rev. Lett. **109**, 166804 (2012).
[5] J. S. Schoenfield, B. M. Freeman, and H. Jiang, Nature Commun. **8**, 64 (2017).
[6] B. Thorgrimsson, D. Kim, Y.-C. Yang, L. Smith, C. Simmons, D. R. Ward, R. H. Foote, J. Corrigan, D. Savage, M. Lagally, M. Friesen, S. Coppersmith, and M. Eriksson, npj Quant. Inform. **3**, 32 (2017).
[7] X. Mi, S. Kohler, and J. Petta, Phys. Rev. B **98**, 161404 (2018).
